# Serum MUC5AC protein levels are correlated with the development and severity of connective tissue disease-associated pulmonary interstitial lesions

**DOI:** 10.3389/fimmu.2022.987723

**Published:** 2022-09-15

**Authors:** Lin Weng, Wei Liu, Lingye Wang, Zhichao Wu, Dehao Liu, Yihua Lin, Shuli Song, Ciyuan Yu, Yaqiong Chen, Juan Chen, Shengxiang Ge

**Affiliations:** ^1^ Department of Rheumatology, The First Affiliated Hospital of Xiamen University, School of Medicine, Xiamen University, Xiamen, China; ^2^ State Key Laboratory of Molecular Vaccinology and Molecular Diagnostics, National Institute of Diagnostics and Vaccine Development in Infectious Disease, Collaborative Innovation Centers of Biological Products, School of Public Health, Xiamen University, Xiamen, China; ^3^ Department of Radiology, The First Affiliated Hospital of Xiamen University, School of Medicine, Xiamen University, Xiamen, China; ^4^ Department of Respiratory Medicine, The First Affiliated Hospital of Xiamen University, School of Medicine, Xiamen University, Xiamen, China

**Keywords:** connective tissue disease, interstitial lung disease, mucin 5AC, mucin 5B, Sjögren’s syndrome, systemic sclerosis, polymyositis (PM), dermatomyositis (dm)

## Abstract

**Background:**

Mucin 5AC (MUC5AC) and mucin 5B (MUC5B) are the major components of airway mucins. The expression levels of MUC5AC and MUC5B are related to connective tissue disease-associated interstitial lung disease (CTD-ILD) in the promoter region of MUC5AC and MUC5B and the relevant bronchoalveolar lavage fluid. However, the serum protein levels of MUC5AC and MUC5B have not been tested in CTD-ILD patients. In this study, we tested the serum levels of MUC5AC and MUC5B proteins in CTD-ILD patients and assessed their relationship with the occurrence and development of ILD.

**Methods:**

Serum samples were obtained from 168 CTD and 80 healthy participants from the First Affiliated Hospital of Xiamen University. The serum levels of MUC5AC and MUC5B proteins were measured by enzyme-linked immunosorbent assay.

**Results:**

Of the 168 individuals with CTD, 70 had primary Sjögren’s syndrome (pSS), 64 had systemic sclerosis (SSc), and 34 had polymyositis/dermatomyositis (PM/DM). There were 116 cases with concurrent ILD; ILD scores were 1 (n=23), 2 (n=41), and 3 (n=52). Serum MUC5AC and MUC5B protein levels were considerably higher in CTD-ILD than CTD-only individuals or healthy controls (both *p*<0.005). Among the CTD subgroups, MUC5AC was higher in individuals with concurrent ILD than in those without ILD (all *p*<0.05). MUC5AC was positively correlated with ILD severity in all three CTD subgroups (all R>0.47 and all *p*<0.05). The MUC5B levels varied substantially between SSc and SSc patients with concurrent ILD (*p*=0.032) and were related to ILD severity only in PM/DM patients (R=0.346 and *p*=0.045).

**Conclusion:**

MUC5AC is correlated with the occurrence and development of ILD, while MUC5B is associated with ILD diagnosis and severity in CTD subgroups. Serum MUC5AC levels present a definite diagnostic utility for CTD-ILD and as proxies for its severity.

## Introduction

Connective tissue disease (CTD) refers to various autoimmune diseases targeting connective tissues, commonly resulting in collagen deposition, tissue destruction, and ultimately leading to target organ failure (1). Interstitial lung diseases (ILD) are the most common complications of CTD, occurring in approximately 10–50% of CTD patients (2). CTD-ILD mainly includes rheumatoid arthritis associated ILD (RA-ILD), systemic sclerosis associated ILD (SSc-ILD), primary Sjögren’s syndrome associated ILD (pSS-ILD), and polymyositis/dermatomyositis associated ILD (PM/DM-ILD) (3–6). CTD-ILD is a more severe disease than CTD, as individuals with RA-ILD show a rate of mortality that is 2–10 times higher than RA patients without ILD (7).

The pathophysiology of CTD-ILD is unclear but genetic factors are thought to play an essential role in its development (8). Previous studies have demonstrated a strong relationship between ILDs and single nucleotide polymorphisms (SNPs) in the promoter regions of mucin 5B (MUC5B) (7, 9–11) and mucin 5AC (MUC5AC) (12). MUC5AC and MUC5B, which account for approximately 90% of the mucoproteins (13), play a crucial role in the respiratory mucosal defense arsenal. Li et al. found that in ILD patients, levels of MUC5AC protein were significantly higher in bronchoalveolar lavage fluid (BALF) than in pleural effusions; these levels were associated with the severity of pulmonary lesions. However, serum is a more readily accessible biofluid than BALF in clinical practice. Meng et al. reported that human epididymal protein 4 (HE4) expression was higher in the serum and lung tissues of CTD patients who had interstitial pneumonia vulgaris (UIP). Furthermore, serum HE4 was suggested to be a potential biomarker for measuring illness severity and predicting the prognosis of UIP-CTD patients (14). Prior research has primarily focused on the KL-6 protein in the MUC1 family, which is present at higher levels in the serum of ILD patients (15). However, there are no reports on MUC5AC and MUC5B protein levels in the serum of CTD-ILD patients. Given this, in the present study, we recruited CTD patients with concurrent pSS, SSc, or PM/DM to quantify the serum levels of MUC5AC and MUC5B proteins and to compare the differences in expression among individuals with CTD, healthy controls, and individuals with CTD and concurrent ILD. We further evaluated the trends of MUC5AC and MUC5B in patients with different ILD grades and explored the relationship between these proteins and ILD severity in CTD patients.

## Methods

### Samples

The study was approved by the Ethics Committee of the First Affiliated Hospital of Xiamen University (Ethics No.: KY2017-026). Participants provided written informed consent before participation in the study. Between February 2020 and December 2021, we recruited 168 CTD patients and 80 healthy participants from the First Affiliated Hospital of Xiamen University. The CTD group included individuals with pSS, SSc, and PM/DM. The diagnoses of pSS and SSc were based on the relevant criteria of the American College of Rheumatology (ACR) and the European League Against Rheumatism (EULAR) (16, 17). PM/DM patients were diagnosed according to European Neuromuscular Centre criteria. ILD diagnosis and grading among CTD patients were conducted using the American Thoracic Society (ATS) and European Respiratory Society (ERS) criteria (18). High resolution computed tomography (HRCT) scan-based classification was assessed by three independent reviewers (blinded to the individual’s clinical data) with the following grading scheme, which was assigned a numerical score based on the type and distribution of interstitial lung abnormalities that consisted of septal lines, reticulation, traction bronchiectasis, cyst formation, and/or ground glass opacification: 0=non ILD; 1=indeterminate ILD (focal or unilateral ground-glass attenuation, focal or unilateral reticulation, or patchy ground-glass abnormalities involving <5% of the lung); 2=mild/early ILD (changes affecting >5% of any lobar region with non-dependent ground-glass or reticular abnormalities, diffuse centrilobular nodularity, non-emphysematous cysts, honeycombing, or traction bronchiectasis); and 3=advanced ILD (bilateral fibrosis in multiple lobes associated with honeycombing and traction bronchiectasis in a subpleural distribution) (19–21). Contradictory gradings were repeated and then resolved *via* consensus; final scores ≥1 were classified as ILD. Due to the COVID-19 outbreak, pulmonary function testing was examined in only a little fraction of patients. Thus all participants were graded for ILD severity by a chest HRCT for this study. Peripheral blood was collected in coagulating tubes, the blood placed at room temperature for 30min and centrifuged at 1500g for serum collection, and immediately frozen at −80°C.

### Enzyme-linked immunosorbent assay

Serum MUC5AC and MUC5B protein levels were measured with double-antibody sandwich ELISA (Novus Biologicals, USA; NBP2-76703 and NBP2-76705) according to the manufacturer’s instructions. Briefly, samples and standards were diluted tenfold with sample diluent, added in 100 μL volumes to the microplate, and then incubated at 37°C for 90 minutes.in which a single-well assay conducted the samples, and the standards were tested in triplicates. Biotin-labeled antibody solution was added to the samples and incubated at 37°C for 60 minutes. After washing, a solution labeled with horseradish peroxidase streptavidin was added and incubated at 37°C for 30 minutes. After washing, TMB substrate solution was added and incubated at 37°C for 15 minutes, then terminated with a stop solution. Readings were acquired at 450 nm and 630 nm using a microplate reader (Antubio Diagnostics Co., Ltd., Zhengzhou) then the MUC5AC and MUC5B protein concentrations were calculated using wavelength-corrected optical densities and the standard curve readings.

### Statistical analysis

Statistical analyses were performed using IBM SPSS version 25.0 (Armonk, NY) and plots were generated using Graphpad Prism 8 (Graphpad Software Corp.). Based on the normality and homogeneity of variance of the data, between-group differences in continuous variables were tested by using Student’s t-test or the Mann-Whitney U test. Categorical variables were tested by using the Chi-square test and Fisher’s exact test. Statistical significance was determined using two-tailed tests and p<0.05.

## Results

### Characteristics of the study population

168 CTD and 80 healthy participants were recruited. The CTD group included 35 males and 133 females with a sex ratio of 0.263 which was significantly lower than in healthy controls (26/54, *p*=0.046). The median age was 42 years (29.25–54.75 years) in CTD and 53 years (43–61 years) in healthy controls (*p*=0.002).

The CTD group included 70 pSS, 64 SSc, and 34 PM/DM cases. Among these CTD subgroups, ILD scores were distributed as follows: 0 (n=30), 1 (10), 2 (15), and 3 (15) in pSS; 0 (15), 1 (10), 2 (15), and 3 (24) in SSc; and 0 (7), 1 (3), 2 (11), and 3 (13) in PM/DM. Demographic and CTD characteristics and autoantibody detection results of patients with pSS, SSc, and PM/DM are listed in **Table 1**, most of which were not substantially different among the distinct ILD severity scores. Only age, gender, and the anti-SSB positive rate of pSS patients and the positivity rate of myositis-specific antibodies (MSAs) in PM/DM patients were statistically different between the different ILD scores, with *p*-values of 0.011, 0.007, 0.017, and 0.041(**Table 1**).

**Table 1:** Characteristics of the CTD patients with different ILD severity.

	*No ILD (ILD score 0)*	*Indeterminate ILD (ILD score 1)*	*Mild/moderate ILD (ILD score 2)*	*Severe ILD (ILD score 3)*	*P value*
***pSS*** *(n=70)*					
*Demographic characteristics*					
*Number of individuals*	30	10	15	15	
*Sex (male: female)*	0.00 (0:30)	0.11 (1:9)	0.15(2:13)	0.50 (5:10)	0.011^*^
*Age (median, range)*	45.67 (30.0-57.25)	52.5 (46.00-61.00)	62.4(56.00-68.00)	61.47 (55.0-68.0)	0.007^**^
*Disease duration (IQR), years*	2.05(1.00-5.25)	1.50(0.63-5.38)	5.00(2.00-10.00)	2.00(1.00-4.20)	0.593
*pSS characteristics*					
*labial glands biopsy, positive. (%)*	14(46)	3(30)	4(27)	6(40)	0.503
*xerophthalmia, positive. (%)*	13(43)	2(20)	4(27)	6(40)	0.477
*Autoantibodies*					
*Antinuclear antibody, positive. (%)*	27 (90)	8 (80)	13 (87)	14 (94)	0.759
*Anti-SSA/Ro, positive. (%)*	24 (80)	7(70)	8(54)	9 (60)	0.266
*Anti-SSB/La, positive. (%)*	18 (60)	1 (10)	4(27)	5 (33)	0.017*
*Anti Ro-52, positive. (%)*	21(70)	6(60)	11 (73)	12(80)	0.744
***SSc*** *(n=64)*					
*Demographic characteristics*					
*Number of individuals*	15	10	15	24	
*Sex (male: female)*	0.15 (2:13)	0.67 (4:6)	0.15 (2:13)	0.26 (5:19)	0.347
*Age (median, range)*	43.26 (31.00-60.00)	49.40 (41.75-56.75)	47.26(42.00-56.00)	50.67 (43.25-60.00)	0.524
*Disease duration (IQR), years*	1.50(0.50-4.00)	2.50(0.31-6.25)	2.00(2.00-7.00)	4.43(2.00-9.50)	0.534
*SSc characteristics*					
*Raynaud phenomenon, positive (%)*	6(40)	5(50)	8(53)	13(54)	0.880
*Finger swelling, positive. (%)*	0(0)	1(10)	2(13)	7(29)	0.093
*scleroderma, positive. (%)*	0(0)	1(10)	2(13)	3(12)	0.932
*Autoantibodies*					
*Antinuclear antibody, positive. (%)*	12 (80)	9(90)	14 (94)	23 (96)	0.408
*Anti Scl-70, positive. (%)*	8(53)	6(60)	6 (40)	16 (67)	0.427
*Anti CENP B, positive. (%)*	1 (7)	1 (10)	5 (34)	6 (25)	0.438
***DM/PM*** *(n=34)*					
*Demographic characteristics*					
*Number of individuals*	7	3	11	13	
*Sex (male: female)*	1.33(4:3)	0.5 (1:2)	1.75(7:4)	0.18(2:11)	0.083
*Age (median, range)*	51.0 (39.0-69.0)	56 (41.00-57.50)	55.6 (52.0-60.0)	59.3(48.0-68.0)	0.822
*Disease duration (IQR), years*	0.50(0.25-1.00)	0.5(0.3-5.25)	1.20(0.17-3.00)	2.00(1.20-4.00)	0.773
*DM/PM characteristics*					
*Gottron sign, positive. (%)*	3(43)	1(33)	5(45)	4(31)	0.886
*Heliotrope sign, positive. (%)*	2(29)	1(33)	5(45)	4(31)	0.860
*Amyasthenia, positive. (%)*	3(43)	1(33)	5(45)	3(23)	0.676
*Autoantibodies*					
*myositis specific antibody, positive. (%)*	7(100)	2(67)	11(100)	7(57)	0.041*

SSc, systemic sclerosis; pSS, primary Sjogren’s syndrome; DM, dermatomyositis; PM, polymyositis; *p < 0.05, ^**^p < 0.01 when compared with the severity of ILD and the factors was compared, Chi-square test were used to find p values were presented.

### Serum MUC5AC levels in CTD patients with and without ILD

**Figure 1:**
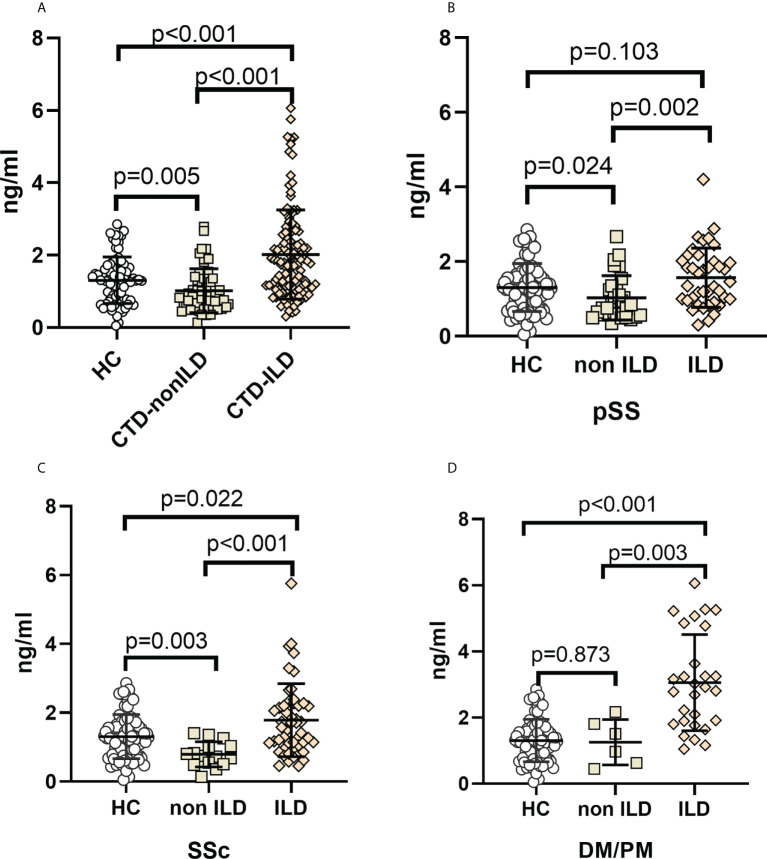
Elevation of MUC5AC levels in CTD-ILDs **(A).** CTD-ILD, CTD-non ILD, and healthy controls. **(B)**. pSS-ILD, pSS-non ILD, and healthy controls. **(C).** SSc-ILD, SSc-non ILD, and healthy controls. **(D)**. DM/PM-ILD, DM/PM-non ILD, and healthy controls. Dot plots depict levels (measured by standard solid-phase enzyme-linked immunosorbent assay) of MUC5AC in individual serum samples from patients without ILD (interstitial lung abnormality ILD score 0), patients with ILD (indeterminate ILD (ILD score 1), mild ILD (ILD score 2), and advanced ILD (ILD score 3)) and healthy controls, Each symbol represents an individual patient; horizontal lines show the mean. p values were determined by Mann-Whitney U test.

Median MUC5AC levels were 1.30 ng/mL (IQR, 0.75 to 1.63 ng/mL) in the healthy controls, 0.84 ng/mL (IQR, 0.56 to 1.34 ng/mL) in the CTD-non ILD group, and 1.80 ng/mL (IQR, 1.13 to 2.60 ng/mL) in the CTD-ILD group. Serum MUC5AC was significantly higher in the CTD-ILD than in the CTD-non ILD (*p*<0.001) or healthy control (*p*<0.001) groups. Serum MUC5AC was slightly lower in the CTD-non ILD group than in healthy controls (*p*=0.005) (**Figure 1A**). When evaluating patients with different types of CTD (**Figures 1B–D**), serum MUC5AC was higher in the pSS-ILD, SSc-ILD, and PM/DM-ILD subgroups than in the respective non-ILD group (*p*=0.002, *p*<0.001, and *p*=0.003, respectively). Compared to the healthy control group, serum MUC5AC was much higher in the SSc-ILD (*p*=0.022) and PM/DM-ILD (*p*<0.001) groups but not in the pSS-ILD group (*p*=0.103). Compared to the healthy controls, serum MUC5AC levels were lower in the pSS-non ILD (*p*=0.024) and SSc-non ILD (*p*=0.003) groups but not in the PM/DM-non ILD group (*p*=0.873).

### Serum MUC5B levels in CTD patients with and without ILD

**Figure 2:**
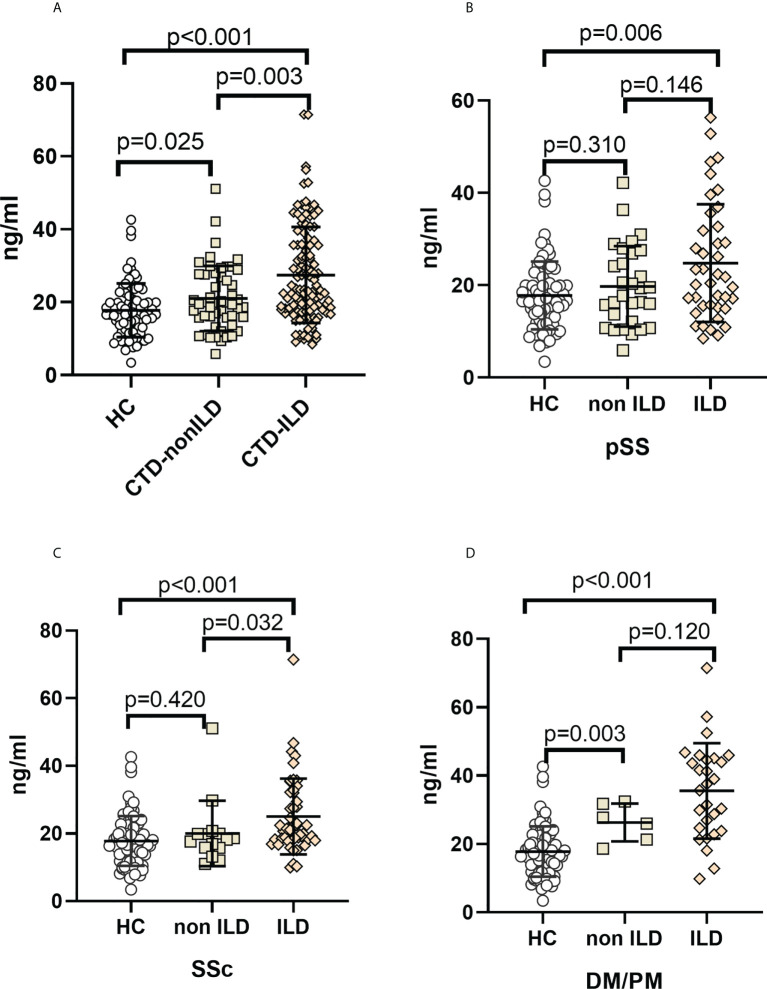
Elevation of MUC5B levels in CTD-ILDs **(A)**. CTD-ILD, CTD-non ILD, and healthy controls. **(B).** pSS-ILD, pSS-non ILD, and healthy controls. **(C).** SSc-ILD, SSc-non ILD, and healthy controls. **(D).** DM/PM-ILD, DM/PM-non ILD, and healthy control. The methodology used for these experiments was identical to those described in the legend for **Figure 1**.

Median serum MUC5B protein levels were 16.62 ng/mL (IQR, 12.57 to 21.48 ng/mL) in healthy controls, 19.68 ng/mL (IQR, 15.75 to 27.67 ng/mL) in CTD-non ILD, and 23.25 ng/mL (IQR, 17.84 to 35.70 ng/mL) in CTD-ILD. Unlike MUC5AC, serum MUC5B levels were elevated in CTD-ILD patients compared to CTD-non ILD patients (*p*=0.004) and healthy controls (*p*<0.001). CTD-non ILD patients also showed higher MUC5B compared to healthy controls (*p*=0.025) (**Figures 2A**). As shown in **Figures 2B–D**, compared to healthy controls, serum MUC5B levels were higher in pSS-ILD (*p*=0.006), SSc-ILD (*p*<0.001), and PM/DM-ILD (*p*<0.001). Serum MUC5B levels were higher in SSc-ILD compared to SSc-non ILD (*p*=0.032) and were considerably elevated in PM/DM-non ILD compared to healthy controls (*p*=0.003).

### Correlation between serum MUC5AC/MUC5B and ILD severity

**Figure 3:**
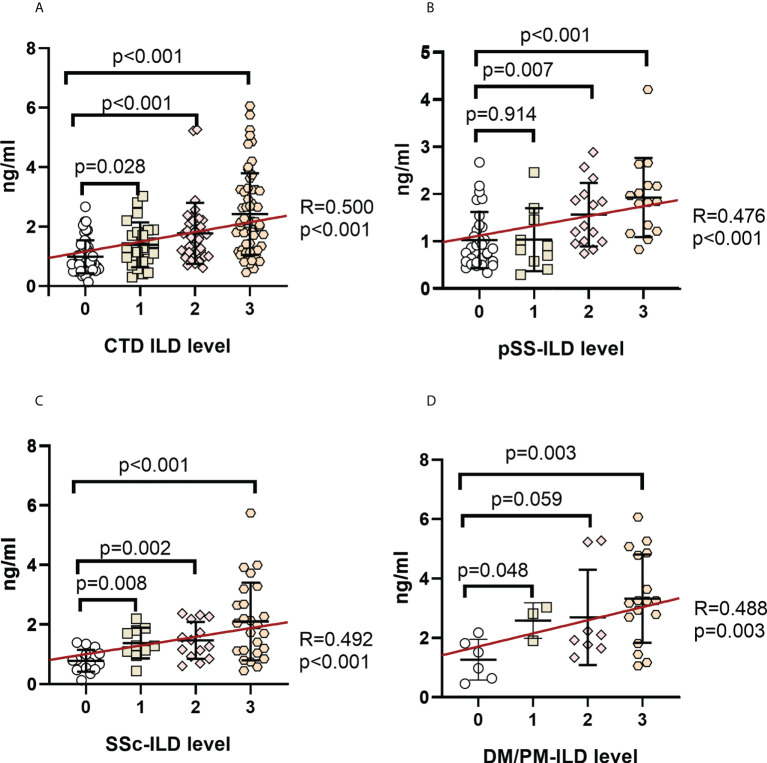
Correlation between serum MUC5AC levels and ILD severity in patients with various autoimmune disease(s) **(A)** CTD-ILD. **(B).** pSS-ILD. **(C).** SSc-ILD. **(D).** DM/PM-ILD. Dot plot depicts the correlation between the level of MUC5AC and the severity of ILD in individual serum samples from patients with no ILD (interstitial lung abnormality (ILD score 0), uncertain ILD (ILD score 1), mild ILD (ILD score 2), or advanced ILD (ILD score 3) (measured by standard solid-phase enzyme-linked immunosorbent assay). Each symbol represents an individual patient; horizontal lines show the mean. Categorical variables were tested by using the Chi-square test and Fisher’s exact test. Statistical significance was determined using two-tailed tests and p<0.05.

**Figure 4:**
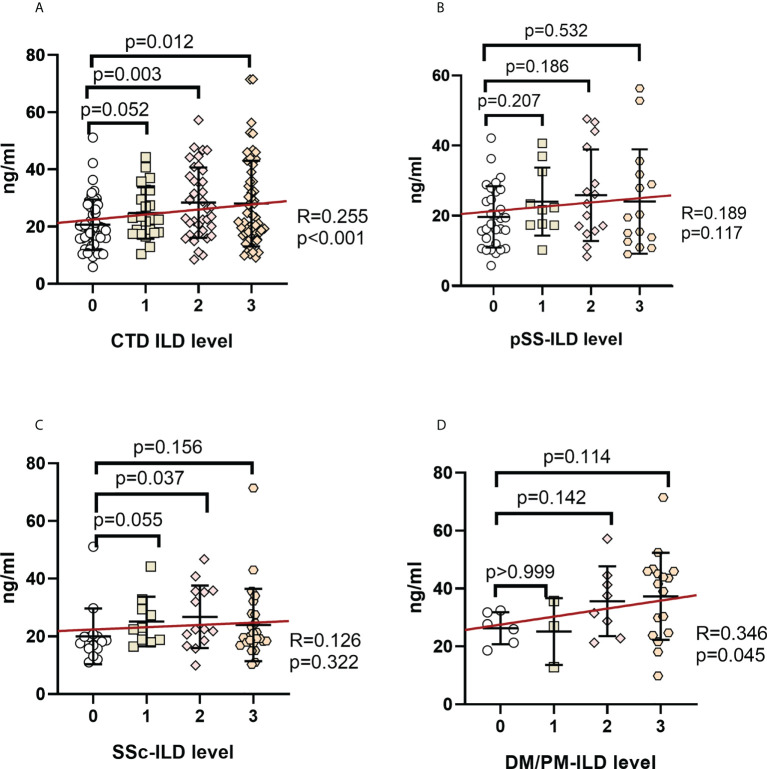
Correlation between serum MUC5B levels and ILD severity in patients with various autoimmune disease(s) **(A)**. CTD-ILD. **(B).** pSS-ILD. **(C).** SSc-ILD. **(D)**. DM/PM-ILD. The methodology used for these experiments was identical to those described in the legend for **Figure 3**.

Serum MUC5AC and MUC5B levels among the ILD grades in CTD patients were compared to investigate their association with ILD severity (**Figures 3A**, **4A**). Serum MUC5AC and MUC5B were found to be positively correlated with ILD severity. The correlation between ILD scores and serum MUC5AC (R=0.500) was more substantial than with MUC5B (R=0.255). When considering the different forms of CTDs, serum MUC5AC levels were closely related to ILD severity (**Figures 3B–D**); pSS (R=0.476), SSc (0.492), and PM/DM (0.488). However, the correlation between serum MUC5B and ILD severity was lower in pSS (R=0.189) and SSc (R=0.126; **Figures 4B, C**), but was slightly higher in PM/DM (R=0.346; **Figure 4D**).

## Discussion

ILD is a serious complication of CTD and is the leading cause of CTD-associated deaths. ILD complications occur in around 35–52% of SSc (22), 6–94% of pSS (23), and 35–48% of PM/DM (24) patients. The mortality associated with SSc, pSS, and DM with ILD is significantly higher than in CTDs without ILD (23, 25, 26). Although disease severity has been linked with SNPs in the promoter region of MUC5AC and MUC5B, which are the most abundant airway mucins (7, 9–12), and MUC5AC protein levels in BALF (27), there have been no reports on the serum levels of MUC5AC and MUC5B in CTD-ILD patients.

Due to the lack of simple laboratory diagnostic targets, the diagnosis of ILD still relies on high-resolution computed tomography (HRCT) and lung function, which is influenced by clinicians’ experience and highly subjective. Therefore, a rapid and simple laboratory index is urgently needed for the clinical differential diagnosis of ILD. We found that serum MUC5AC and MUC5B are significantly increased in individuals with CTD-ILD compared to those without ILD complications or healthy controls. We also found that the serum levels of these proteins were positively correlated with ILD severity. It indicates that both MUC5AC and MUC5B may be potential biomarkers of ILD, so it may provide a simple detection method for the diagnosis of ILD and assess its severity.

In the three CTD subgroups, serum MUC5AC was higher in patients with concurrent ILD than in those without concurrent ILD (**Figures 1B–D**). Considering the correlation with the severity stratification of concurrent ILD in CTD patients, there was a closer relationship between the ILD score and serum MUC5AC than MUC5B.

While serum MUC5B was found to be higher in SSc-ILD than in SSc-non ILD groups (**Figure 2C**), In contrast, MUC5B was only associated with the ILD score in the PM/DM group (R=0.346, *p*=0.045). These suggest that MUC5B is not highly correlated with ILD severity in CTD, despite being much higher in CTD-ILD than in CTD-non ILD; this is consistent with the results of Lee et al. Lee et al. also showed that the rs35705950 SNP in the MUC5B promoter region is associated with idiopathic pulmonary fibrosis but has no apparent connection with CTD-ILD. Therefore, MUC5B may be able to distinguish ILD lesions caused by different CTDs. For example, DM/PM-ILD has a significant correlation with MUC5B levels.

Because MUC5AC has a significant correlation with the severity of ILD, with the increase of ILD severity, the expression level of MUC5AC tends to rise. And by analyzing the differential ability of MUC5AC between CTD-non ILD and CTD-ILD, the AUC value of MUC5AC reached 0.78, which has a good ability to distinguish whether CTD patients have ILD disease, While the AUC value of MUC5B was only 0.68(**Supplementary Figure 1**). So we also found that the tested serum protein levels were distinctly different among the CTD subgroups when complicated by ILD, suggesting that there might exist some variances in the mechanisms of ILD development within the cohort. Given the patients with three clinical subtypes, serum MUC5AC and MUC5B levels were significantly higher in SSc-ILD than SSc, and MUC5AC was higher in pSS-ILD and PM/DM-ILD than in those without ILD. Serum MUC5AC and MUC5B were increased with ILD severity in PM/DM, whereas only MUC5AC protein levels increased with ILD scores in the pSS and SSc groups. These results suggest that we need to evaluate different biomarkers for ILD severity when considering different CTDs.

There were some limitations of this study. First, we recruited only individuals with pSS, SSc, and PM/DM. Whether serum MUC5AC levels might better reflect the occurrence and development of ILD needs to be verified in more patients with other CTDs. And when collecting the healthy control group, the problem of age and sex matching was not considered in this study. However, when analyzing the influence of age and sex on the expression level of MUC protein, it was found that there was no significant difference (**Supplementary Figure 2**), so the main conclusions of this study were not affected. Smoking is a problem of great concern in ILD. However, only 4 patients with smoking history were found in this study, which may be due to that the majority of patients were female (78%). In China, most women do not smoke. So, the impact of smoking on MUC5AC levels cannot be analyzed. In addition, some clinical indicators or biomarkers, such as BMI, eGFR, CRP, etc., were not collected due to the study design, which made it impossible to conduct a more comprehensive analysis on their influence. Furthermore, the clinical samples were relatively small, especially low-grade ILD. Additional work is required to validate the utility of serum MUC5AC in CTDs. And whether MUC5AC can be used as a monitoring indicator for the treatment of ILD patients’ needs to be further tested and evaluated by collecting the sample volume of follow-up patients.

In general, serum MUC5AC protein levels were considerably higher in individuals with CTD-ILD than in those without ILD. Thus, MUC5AC is a potential biomarker for predicting ILD progression. The levels of MUC5AC in the peripheral blood are much higher expression that may be used as a biomarker target for the early detection and treatment of CTD-ILD patients.

## Data availability statement

The original contributions presented in the study are included in the article/**Supplementary Material**. Further inquiries can be directed to the corresponding authors.

## Ethics statement

The studies involving human participants were reviewed and approved by ethics committee of the First Affiliated Hospital of Xiamen University (Ethics No.: KY2017-026). The patients/participants provided their written informed consent to participate in this study.

## Author contributions

JC and SG conceived the study. JC acquired funding. LW and WL collected the data, conducted data analysis, and drafted manuscript. LW, WL, L-YW, ZW, DL, YL, SS, CY, and YC performed laboratory tests. JC and SG were responsible for supervision of the study. All authors critically reviewed the manuscript and approved the final version.

## Funding

This study was funded by the National Natural Science Foundation of China (Grant No. 81771751) to JC and Fujian Provincial Department of Science and Technology (Grant No. 2018J01383) to JC.

## Acknowledgments

The authors would like to express their gratitude to EditSprings (https://www.editsprings.com/) for the expert linguistic services provided.

## Conflict of interest

The authors declare that the research was conducted in the absence of any commercial or financial relationships that could be construed as a potential conflict of interest.

## Publisher’s note

All claims expressed in this article are solely those of the authors and do not necessarily represent those of their affiliated organizations, or those of the publisher, the editors and the reviewers. Any product that may be evaluated in this article, or claim that may be made by its manufacturer, is not guaranteed or endorsed by the publisher.
