# Supplementary material for: Serum MUC5AC protein levels are correlated with the development and severity of connective tissue disease-associated pulmonary interstitial lesions
Source: Front Immunol. 2022 Sep 15;13:987723. doi: 10.3389/fimmu.2022.987723 (PMC9520158; doi:10.3389/fimmu.2022.987723)
Supplement: Supplementary file 1 [file Image_1.pdf]

Supplementary Figure 1

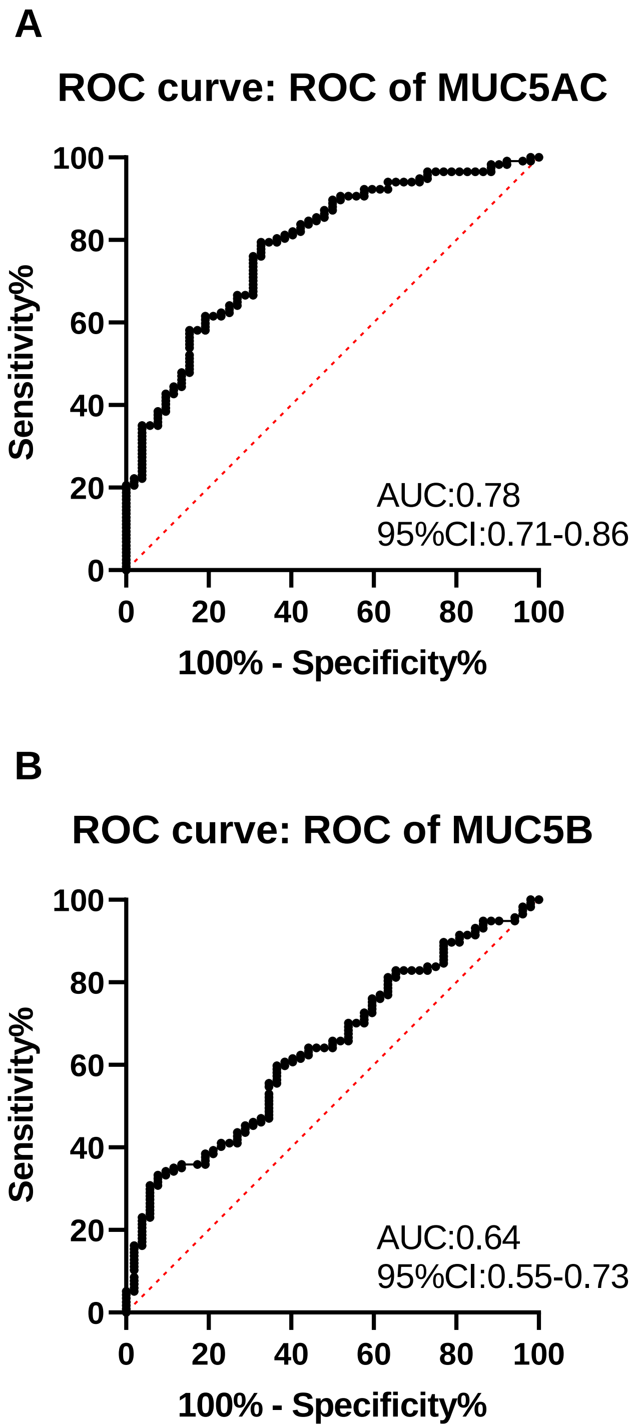

Supplementary Figure 1 : MUC5AC has the potential to diagnose CTD-ILD, and the AUC value of ROC curve for diagnosis of CTD-ILD was 0.78. ROC curves of MUC5AC(**A**) and MUC5B(**B**) for the diagnosis of CTD-non ILD and CTD-ILD.

Supplementary Figure 2

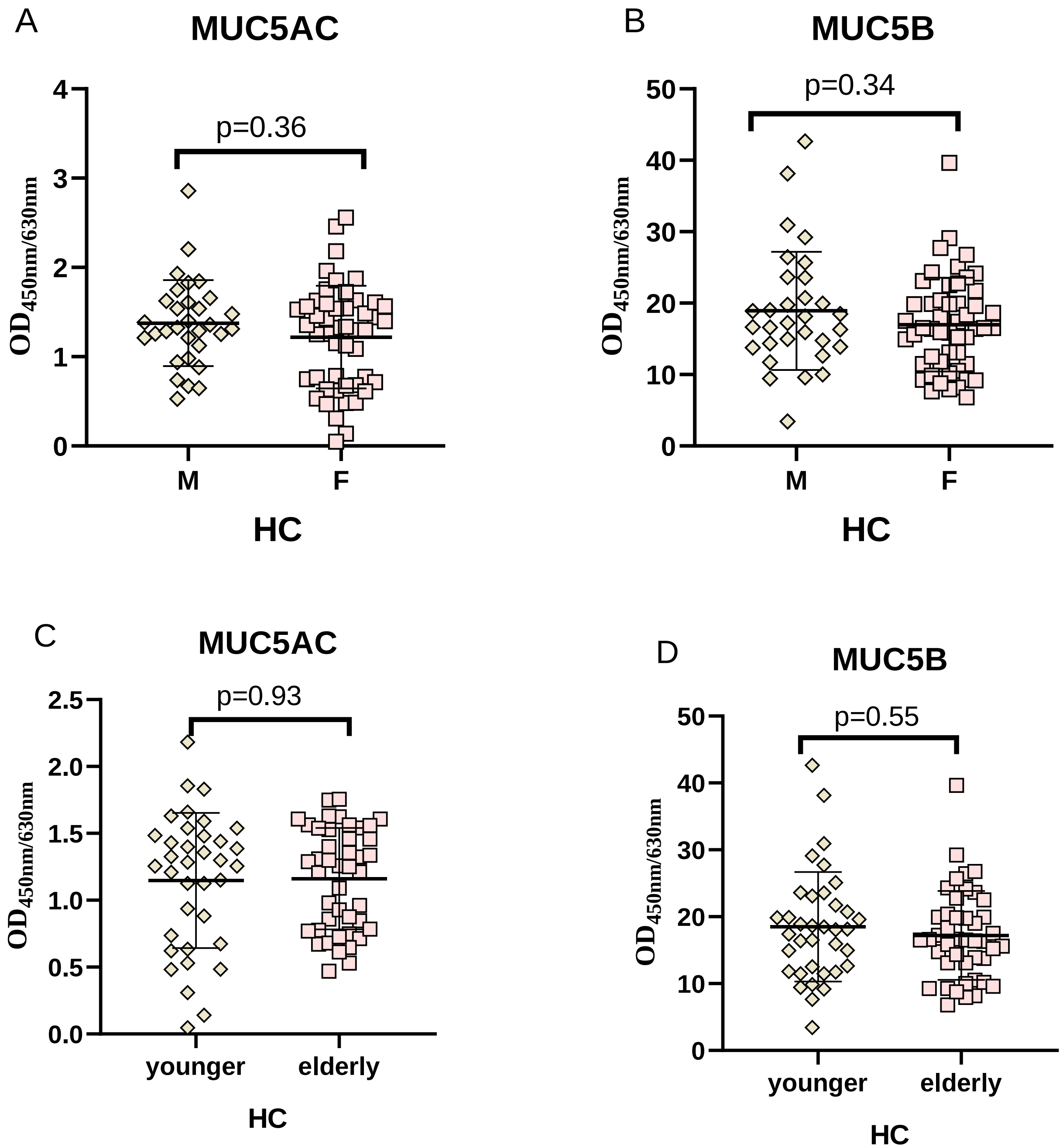

Supplementary Figure 2 : In the healthy controls, the expression levels of MUC5AC and MUC5B were not affected by age and sex. *Age*: **A** : MUC5AC **B** : MUC5B. *Sex*: **C** : MUC5AC **D** : MUC5B. Each symbol represents an individual patient; horizontal lines show the mean. p values were determined by Mann-Whitney U test.

M: males F: femals, younger: the middle-aged and young groups, older: the middle-aged and old groups

Supplementary Figure 3

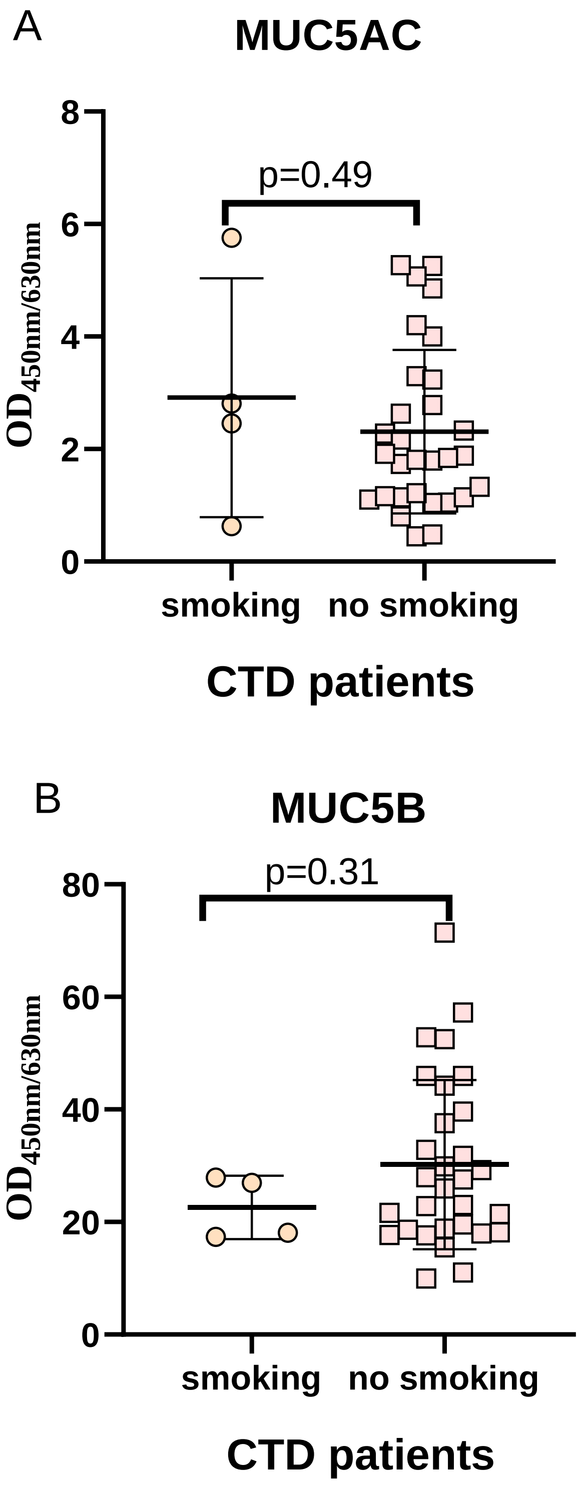

Supplementary Figure 3 : The expression of MUC5AC and MUC5B in CTD patients was not affected by smoking. **A** : MUC5AC **B** : MUC5B. Each symbol represents an individual patient; horizontal lines show the mean. p values were determined by Mann-Whitney U test.
